# Coexistence and Within-Host Evolution of Diversified Lineages of Hypermutable *Pseudomonas aeruginosa* in Long-term Cystic Fibrosis Infections

**DOI:** 10.1371/journal.pgen.1004651

**Published:** 2014-10-16

**Authors:** Sofía Feliziani, Rasmus L. Marvig, Adela M. Luján, Alejandro J. Moyano, Julio A. Di Rienzo, Helle Krogh Johansen, Søren Molin, Andrea M. Smania

**Affiliations:** 1 Centro de Investigaciones en Química Biológica de Córdoba (CIQUIBIC), CONICET, Departamento de Química Biológica, Facultad de Ciencias Químicas, Universidad Nacional de Córdoba, Córdoba, Argentina; 2 Department of Systems Biology, Technical University of Denmark, Lyngby, Denmark; 3 Daphne du Maurier, Centre for Ecology and Conservation, College of Life and Environmental Sciences, University of Exeter, Cornwall Campus, Cornwall, United Kingdom; 4 Estadística y Biometría, Facultad de Ciencias Agropecuarias, Universidad Nacional de Córdoba, Córdoba, Argentina; 5 Department of Clinical Microbiology 9301, Rigshospitalet, Copenhagen University Hospital, Copenhagen, and The Novo Nordisk Foundation Centre for Biosustainability, Hørsholm, Technical University of Denmark, Denmark; Université Paris Descartes, INSERM U1001, France

## Abstract

The advent of high-throughput sequencing techniques has made it possible to follow the genomic evolution of pathogenic bacteria by comparing longitudinally collected bacteria sampled from human hosts. Such studies in the context of chronic airway infections by *Pseudomonas aeruginosa* in cystic fibrosis (CF) patients have indicated high bacterial population diversity. Such diversity may be driven by hypermutability resulting from DNA mismatch repair system (MRS) deficiency, a common trait evolved by *P. aeruginosa* strains in CF infections. No studies to date have utilized whole-genome sequencing to investigate within-host population diversity or long-term evolution of mutators in CF airways. We sequenced the genomes of 13 and 14 isolates of *P. aeruginosa* mutator populations from an Argentinian and a Danish CF patient, respectively. Our collection of isolates spanned 6 and 20 years of patient infection history, respectively. We sequenced 11 isolates from a single sample from each patient to allow in-depth analysis of population diversity. Each patient was infected by clonal populations of bacteria that were dominated by mutators. The *in vivo* mutation rate of the populations was ∼100 SNPs/year–∼40-fold higher than rates in normo-mutable populations. Comparison of the genomes of 11 isolates from the same sample showed extensive within-patient genomic diversification; the populations were composed of different sub-lineages that had coexisted for many years since the initial colonization of the patient. Analysis of the mutations identified genes that underwent convergent evolution across lineages and sub-lineages, suggesting that the genes were targeted by mutation to optimize pathogenic fitness. Parallel evolution was observed in reduction of overall catabolic capacity of the populations. These findings are useful for understanding the evolution of pathogen populations and identifying new targets for control of chronic infections.

## Author Summary

Patients with cystic fibrosis (CF) are often colonized by a single clone of the common, widespread bacterium *Pseudomonas aeruginosa*, resulting in chronic airway infections. Long-term persistence of the bacteria involves the emergence and selection of multiple phenotypic variants. Among these are “mutator” variants characterized by increased mutation rates resulting from the inactivation of DNA repair systems. The genetic evolution of mutators during the course of chronic infection is poorly understood, and the effects of hypermutability on bacterial population structure have not been studied using genomic approaches. We evaluated the genomic changes undergone by mutator populations of *P. aeruginosa* obtained from single sputum samples from two chronically infected CF patients, and found that mutators completely dominated the infecting population in both patients. These populations displayed high genomic diversity based on vast accumulation of stochastic mutations. Our results are in contrast to the concept of a homogeneous population consisting of a single dominant clone; rather, they support a model of populations structured by diverse subpopulations that coexist within the patient. Certain genes involved in adaptation were highly and convergently mutated in both lineages, suggesting that these genes were beneficial and potentially responsible for the co-selection of mutator alleles.

## Introduction

The opportunistic pathogen *Pseudomonas aeruginosa* is found in many environments and can cause acute or chronic infections in a range of hosts from protozoans to plants to humans [1], [2]. In particular, patients with cystic fibrosis (CF) are highly susceptible to chronic colonization by *P. aeruginosa*, which is frequently fatal because of a persistent inflammatory response leading to gradual decline of lung function [3], [4]. In most cases, following a period of recurrent colonizations, a single strain of *P. aeruginosa* becomes predominant and persists for the rest of the patient's life [5], [6]. Genetic adaptation has been shown to play a major role in successful establishment of long-term chronic *P. aeruginosa* infections of CF patients, and natural selection acts on these bacteria in CF airways to accommodate the fixation of mutations that cause beneficial phenotypic changes [7], [8], [9]. The selected phenotypes display traits that differ from those of environmental isolates but are common in populations found in CF patients, suggesting repeatable patterns of long-term adaptation to the CF lung [10], [11], [12].

A trait frequently observed in chronic infections is an increased mutation rate leading to a mutator phenotype [13], [14]. *P. aeruginosa* from chronically infected CF airways was the first natural model to reveal a high proportion of mutators in contrast to reported proportions in acute infections [13]. Hypermutability in CF *P. aeruginosa* is due primarily to inactivation of the mismatch repair system (MRS) through lost function of the antimutator *mutS* and *mutL* genes [15], and 36–54% of CF patients have been shown to be infected by mutator isolates [13], [16], [17], [18], [19]. Theoretical and experimental approaches have attempted to explain the selection of MRS-mutators as the result of co-selection (hitchhiking) with linked beneficial mutations [20], [21], [22], [23], [24], [25], and their overrepresentation as a consequence of high recombination rates [26]. Mutators have been linked to the development of antibiotic resistance both *in vitro* and *in vivo* [13], [27], [28], [29], [30], [31], and have been reported to enhance genetic adaptation to CF airways through increased accumulation of new mutations [32]. However, comparisons between mutators and normo-mutators did not reveal any association between hypermutability and a particular distribution of mutations among genes, even for antibiotic resistance-related genes [32]. No study to date has linked hypermutability in CF adaptation to any specific adaptive mutation [17], [32], [33], nor to any key adaptive trait in the transition to a chronic state of infection [33].

Our previous studies demonstrated a role of MRS deficiency in the acquisition of CF-related phenotypes under *in vitro* conditions such as mucoid conversion [34], *lasR* inactivation [35], [36], and enhanced adaptability in biofilms [37] – all hallmarks of *P. aeruginosa* chronic airway infection. We also reported the ability of MRS deficiency to bias mutagenic pathways toward DNA simple sequence repeats (SSRs), which gave specific mutational spectra under both *in vitro* [34], [38] and *in vivo* conditions [18], [39]. In view of the widespread effect of hypermutability on the process of adaptation to the CF lung [32], it is important to elucidate the evolution of MRS-mutator strains in the course of CF chronic lung infections.

Previous genome analyses of longitudinally collected *P. aeruginosa* from CF patients demonstrated intra-patient genomic diversity of clonal isolates, suggesting that within-host *P. aeruginosa* population dynamics are driven by clonal competition (clonal interference) and/or niche specialization (adaptive radiation) [18], [40], [41], [42]. To further investigate these processes, Chung *et al.* compared the genomes of pairs of randomly selected contemporary isolates sampled from three chronically infected adult CF patients, and found that the pairs were differentiated by 1, 54, and 344 SNPs, respectively. In the latter case, both isolates were mutators [43].

Although mutators are frequently found in CF infections, no whole-genome studies have focused on the within-host evolution of mutators. Similarly, diversity of within-patient pathogen populations is relevant to planning of clinical intervention strategies, elucidation of transmission networks, and understanding of evolutionary processes, but no study to date has involved genome sequencing of a sufficiently large collection of *P. aeruginosa* isolates taken from the same patient at the same time point to facilitate an in-depth analysis of population diversity.

We combined two distinct strategies for a genome-wide analysis of *P. aeruginosa* MRS-mutators: (i) a longitudinal analysis of two separate clonal lineages of mutators obtained from two CF patients; (ii) a within-host population analysis of a large collection of isolates obtained from a single sputum sample from each of the patients, to provide a snapshot of mutator population structure in the CF lung at a single time point. Whole-genome sequencing of 27 *P. aeruginosa* isolates allowed us to quantify the nature and extent of the genomic changes of MRS-mutator clones and provide a panorama of the high genomic diversity that shapes the structure of *P. aeruginosa* mutator populations during long-term adaptation to the CF airway environment.

## Results

### *P. aeruginosa* sample collection

**Figure 1:**
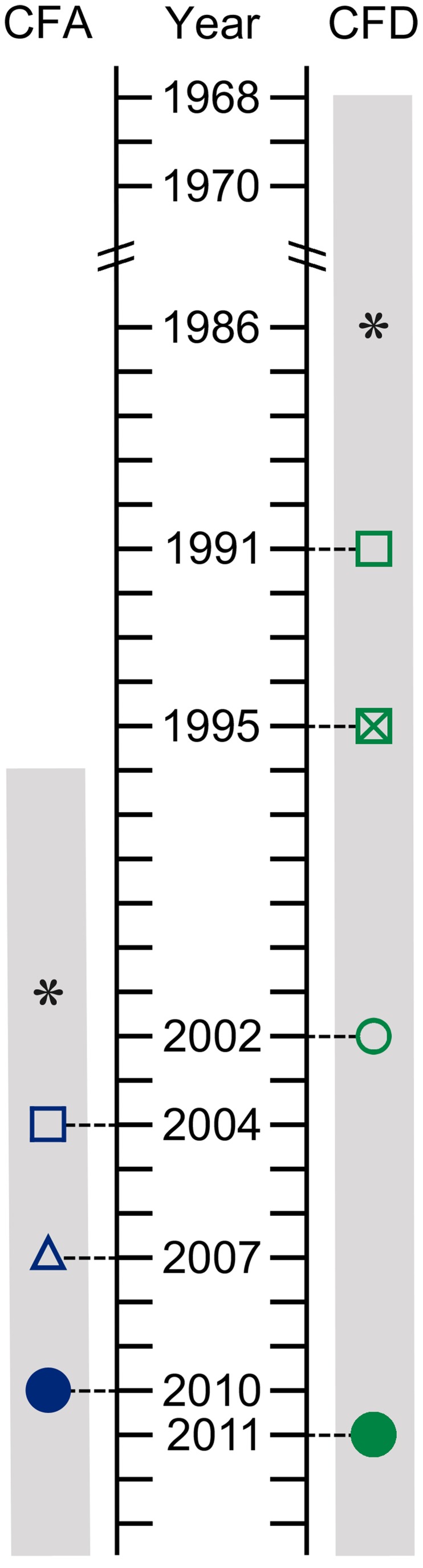
Isolate sampling points and patient life spans. *P. aeruginosa* isolates were collected from two CF patients: CFA and CFD. Hollow symbols: single bacterial isolates. Solid circles: cross-sectional populations of 90 bacterial isolates. *: estimated start of chronic infection. Gray bar: patient life span.

To quantitatively describe the evolutionary processes of MRS-deficient strains during chronic airway infections, we performed longitudinal and cross-sectional analyses of clonal *P. aeruginosa* isolates collected from two CF patients, referred to here as CFA and CFD (Figure 1, and Materials and Methods). The cross-sectional study included 90 isolates obtained from a single sputum sample from each patient. These large collections were used to investigate the clonal genomic diversity within mutator populations in a single host at a single time point. Two different non-epidemic *P. aeruginosa* strains were collected from geographically distant locations, Argentina (CFA) and Denmark (CFD), in which different therapeutic protocols are applied. We were thus able to analyze two independent mutator populations whose evolutionary histories were presumably subjected to common as well as patient-specific selective pressures.

The collection from CFA included: (i) Two sequential isolates obtained in 2004 (CFA_2004/01) and 2007 (CFA_2007/01). These isolates were characterized as MRS-mutators because they harbored missense mutations in the *mutS* and *mutL* genes and showed increased mutation rate (Table 1 and Text S1). (ii) A collection of 90 *P. aeruginosa* isolates obtained from a single sputum sample in 2010 (CFA_2010).

**Table 1:** Mutations in *mutS* and *mutL* genes in *P. aeruginosa* CFA and CFD isolates.

	Isolate	Mutations in MRS genes^a^					Cluster^b^	MRS alleles
		*mutS*			*mutL*			
CFA	2004/01		A739C (T247P)			A1406G (H469R)		SL1
	2007/01		A739C (T247P)			A1406G (H469R)	I	SL1
	2010/40		A739C (T247P)	T2381C (L794P)	T1166C (L389S)	A1406G (H469R)	II	SL2
	2010/31	T478A (W160R)	A739C (T247P)		T1166C (L389S)	A1406G (H469R)	III	SL3
	2010/01	T478A (W160R)	A739C (T247P)		T1166C (L389S)	A1406G (H469R)	III	SL3
	2010/78	T478A (W160R)	A739C (T247P)		T1166C (L389S)	A1406G (H469R)	III	SL3
	2010/82		A739C (T247P)			A1406G (H469R)	IVa	SL1
	2010/43		A739C (T247P)			A1406G (H469R)	IVa	SL1
	2010/72		A739C (T247P)			A1406G (H469R)	IVa	SL1
	2010/87		A739C (T247P)			A1406G (H469R)	IVb	SL1
	2010/32		A739C (T247P)			A1406G (H469R)	IVb	SL1
	2010/26		A739C (T247P)			A1406G (H469R)	IVb	SL1
	2010/11		A739C (T247P)			A1406G (H469R)	IVb	SL1
CFD	1991/01							
	2011/33			−CG at 1551			I	SL1
	2002/01			−CG at 1551			II	SL1
	1995/01			−CG at 1551			III	SL1
	**2011/95**	**+CC at 334**		**−CG at 1551**			**IV**	**SL2**
	**2011/04**	**+CC at 334**		**−CG at 1551**			**IV**	**SL2**
	**2011/45**	**+CC at 334**		**−CG at 1551**			**IV**	**SL2**
	**2011/11**	**+CC at 334**		**−CG at 1551**			**IV**	**SL2**
	**2011/57**	**+CC at 334**		**−CG at 1551**			**IV**	**SL2**
	2011/83			−CG at 1551			V	SL1
	2011/27			−CG at 1551			V	SL1
	2011/34			−CG at 1551			V	SL1
	2011/28			−CG at 1551			VI	SL1
	2011/94			−CG at 1551			VI	SL1

a SNP and indel mutations were considered.

b Clusters were defined according to maximum-parsimony phylogenetic trees (Figure 2).

Amino acid changes are shown in parentheses. Boldface: CFD isolates with reductions in mutation rate. +: insertion. −: deletion.

The collection from CFD included: (i) One normo-mutable isolate obtained in 1991 (CFD_1991/01). (ii) Two sequential MRS-deficient mutators from 1995 (CFD_1995/01) and 2002 (CFD_2002/01) (Table 1 and Text S1) that harbored the same *mutS* missense mutation [33]. (iii) A collection of 90 *P. aeruginosa* isolates obtained from a single sputum sample in 2011 (CFD_2011).

The CFA and CFD collections covered periods of 6 and 20 yrs in the patients' lives, respectively. Based on previous studies indicating a doubling time of 115 min for *P. aeruginosa* in sputum [44], we estimated that ∼36,500 and ∼91,400 duplication events occurred between the first and last isolates collected from CFA and CFD, respectively. Genotypic characterization of the two collections by various molecular methods (Materials and Methods) showed that each patient was chronically infected by a single, unrelated *P. aeruginosa* clone that persisted throughout the study period. The hexadecimal codes [45] for the SNP patterns of the CFA and CFD isolates analyzed are 2C32 and 249A, respectively.

### MRS-deficient mutators are prevalent among CFA and CFD intra-patient *P. aeruginosa* populations

The proportion of MRS-deficient mutators present in each patient was determined based on the rifampicin mutation frequency of the 90 *P. aeruginosa* isolates of the CFA_2010 and CFD_2011 panels. All the CFA_2010 isolates showed mutation frequencies (1.7×10^−5^–7.6×10^−6^) consistent with a strong mutator phenotype. Similarly, in the CFD_2011 panel, strong mutator isolates (1.4×10^−5^–8.3×10^−6^) comprised ∼94% of the population. The remaining 6% showed mutation frequencies close to those observed in the prototypic wild-type normo-mutable strain PAO1 (3×10^−8^–1×10^−7^). These findings were supported by the observed prevalence of mutators in 90 isolates obtained ≥6 months later from new sputum samples (CFA_2011 and CFD_2012); 100% of CFA and 90% of CFD isolates displayed a strong mutator phenotype. To our knowledge, these are the highest proportions of mutators reported to date in large intra-patient populations of *P. aeruginosa* isolated from CF patients. A previous study, which analyzed sets of 40 isolates per sputum sample from 10 CF patients, reported proportions of 27% or less [8]. These findings indicate that *P. aeruginosa* can persist in chronic airway infections without the mutator phenotype, but in certain CF populations, such as those described here, MRS-deficient isolates may be prevalent and dominate the entire infecting population.

### Genomic evolution of *P. aeruginosa* mutator isolates from CF chronic infections

To analyze the genomic evolution of CFA and CFD *P. aeruginosa* mutator lineages, we performed whole-genome sequencing of 13 CFA and 14 CFD isolates. From the CFA collection, we selected and sequenced the initial isolate CFA_2004/01, the intermediate CFA_2007/01, and 11 mutator isolates chosen randomly from the CFA_2010 population (CFA_2010/01, CFA_2010/11, CFA_2010/26, CFA_2010/31, CFA_2010/32, CFA_2010/40, CFA_2010/43, CFA_2010/72, CFA_2010/78, CFA_2010/82, CFA_2010/87). From the CFD collection, we selected and sequenced the initial normo-mutable isolate CFD_1991/01, the intermediate mutators CFD_1995/01 and CFD_2002/01, and 11 isolates from the CFD_2011 population consisting of five normo-mutators (CFD_2011/04, CFD_2011/11, CFD_2011/45, CFD_2011/57, CFD_2011/95) and six randomly selected mutators (CFD_2011/27, CFD_2011/28, CFD_2011/33, CFD_2011/34, CFD_2011/83, CFD_2011/94). The reads of CFA_2004/01 and CFD_1991/01 were assembled *de novo*, yielding genomes of 6,294,248 bp and 6,313,855 bp, respectively (Table S1), which were used as references in subsequent analyses. The sequences of the remaining CFA and CFD isolates were aligned against the corresponding references to assess the genetic changes accumulated in the two mutator lineages during the infection process (Table S2).

Both lineages accumulated a high number of mutations during their evolution in CF airways in comparison with previously reported normo-mutable CF isolates such as the DK2 and PA14 clones [18], [40]. The CFA collection had a total of 2,578 single-nucleotide polymorphisms (SNPs) and 544 1- to 10-bp insertion/deletion mutations (microindels). The CFD collection had a total of 5,710 SNPs and 1,078 microindels (Table S3). We applied Bayesian statistical analysis to infer time-measured phylogenies [46], resulting in estimated mutation rates of 106 SNPs/yr (4.2×10^−9^ SNPs/bp per generation) for CFA and 89 SNPs/yr (3.2×10^−9^ SNPs/bp per generation) for CFD. These findings indicate a mutation rate ∼40-fold higher than that (2.6 SNPs/yr) reported previously for normo-mutable isolates obtained from CF chronic infections [18].

### Genetic diversity generation in *P. aeruginosa* intra-patient mutator populations

SNPs were used as phylogenetic markers to perform a maximum-parsimony reconstruction of the evolutionary relationship of CFA and CFD isolate groups, and to evaluate temporal changes in their population genetic structure (Figure 2). Alleles of *P. aeruginosa* reference strain PAO1 were used to root the trees. In both cases, essentially all SNPs (>99.5%) supported single phylogenetic trees. Interestingly, high genetic diversity was observed in both CFA and CFD *P. aeruginosa* intra-patient populations. CFA_2010 and CFD_2011 contemporary clones were grouped together into three and four distinguishable clusters, respectively: Clusters II, III, and IV for CFA (Figure 2A) and Clusters I, IV, V, and VI for CFD (Figure 2B) trees. Clusters were composed of genetically similar isolates, whereas more extensive genetic dissimilarities were observed between clusters. The branch lengths among clusters differed substantially, indicating uneven mutational loads in coexisting *P. aeruginosa* intra-patient populations.

**Figure 2:**
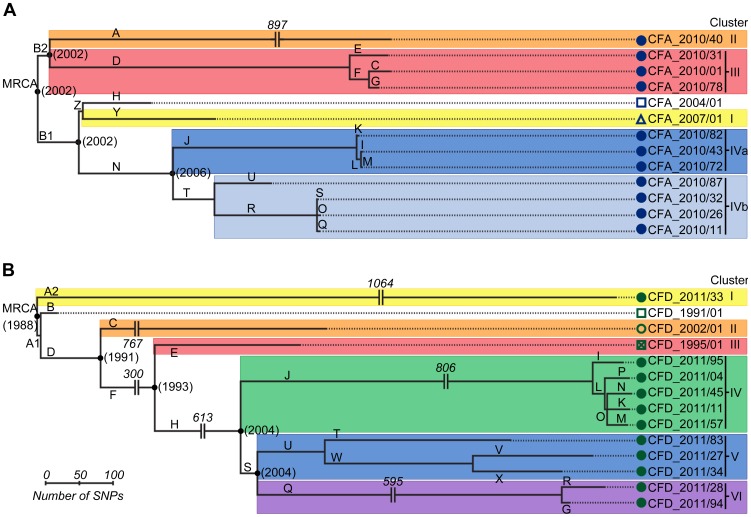
Evolutionary relationships among isolates from CFA and CFD lineages. Maximum-parsimony phylogenetic trees of CFA (A) and CFD (B) were constructed based on the accumulation of new SNPs relative to ancestors CFA_2004/01 and CFD_1991/01. Alleles of *P. aeruginosa* reference strain PAO1 were used to root the trees. Lengths of branches are proportional to the number of accumulated SNPs. Branches are designated by capital letters. MRCA: most recent common ancestor.

All CFD normo-mutable isolates were grouped together in Cluster IV (Figure 2B). In spite of the normo-mutable phenotype, this cluster shared common branches with the intermediate mutator isolates CFD_1995/01 and CFD_2002/01 and with their contemporary mutator clones (branches D, F, and H) (Figure 2B). These findings suggest that the normo-mutators arose from a mutator population of branch J at some point. Cluster IV showed the highest accumulation of mutations during the infection process, despite the low mutation frequencies of its members (Figure 2B and Table S3).

The estimated time points of the most recent common ancestor (MRCA) of the CFA and CFD populations were 2002 and 1988, respectively. Interestingly, each of these estimates coincided with the year at which the patient was diagnosed as chronically infected (Figure 1). This finding indicates that the divergent sub-lineages coexisted for many years – the same as the colonization period of the patient (Figure 2).

**Figure 3:**
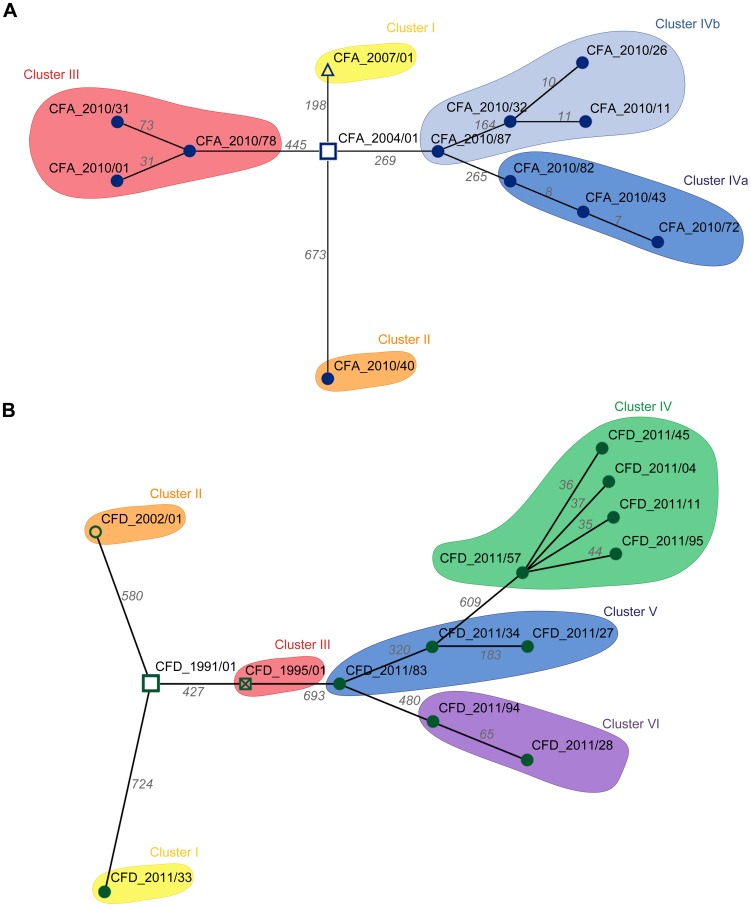
Minimum spanning trees (MSTs) of genomes among CFA and CFD lineages. MSTs for CFA (A) and CFD (B) were constructed based on the total number of genes altered by nonsynonymous SNPs and indel mutations in the respective genomes. Links between nodes represents the minimum distance in terms of mutated genes. Numbers above each link indicate the total amount of mutated genes between the two connected nodes (Table S6). For tree construction, ancestors CFA_2004/01 and CFD_1991/01 were considered as origins.

The question arose whether the genetic diversity observed in the two CF intra-patient populations was associated with differing repertoires of mutated genes among contemporary isolates, or whether most mutations occurred in common genes among the clones. To distinguish between these possibilities, we first determined the set of genes of each CFA and CFD isolate that were altered by nonsynonymous SNPs and microindels. Minimum Spanning Trees (MSTs) were then constructed to illustrate the relationships among contemporary isolates and their corresponding ancestors based on the number of distinctive mutated genes. The CFA_2010 intra-patient *P. aeruginosa* mutator population (Figure 3A) was distributed in three main clusters, whereas the CFD_2011 population (Figure 3B) showed four clusters with all normo-mutable isolates grouped together. The structures of the two MSTs indicate high genetic diversification and a scenario in which even contemporary CFA and CFD mutator populations diversified through mostly different evolutionary pathways.

### Pathoadaptive genes and evidence for parallel evolution in mutator populations

We classified the SNPs according to their distribution in coding and noncoding regions and their effect in translation, to assess the selective forces acting on the CFA and CFD *P. aeruginosa* lineages. Most of the SNPs in CFA and CFD were found to occur within coding regions, and 58.0% and 60.2% (respectively) corresponded to missense mutations (Figure S1). The rates of nonsynonymous to synonymous mutations (dN/dS) were 0.68 for CFA and 0.79 for CFD lineages. Thus, most of the SNPs that became fixed in the *P. aeruginosa* mutator lineages were neutral mutations, with most of each genome showing a signature of purifying selection and/or genetic drift (dN/dS<1; *P* = 5.2×10^−56^ and *P* = 1.5×10^−47^, respectively).

**Figure 4:**
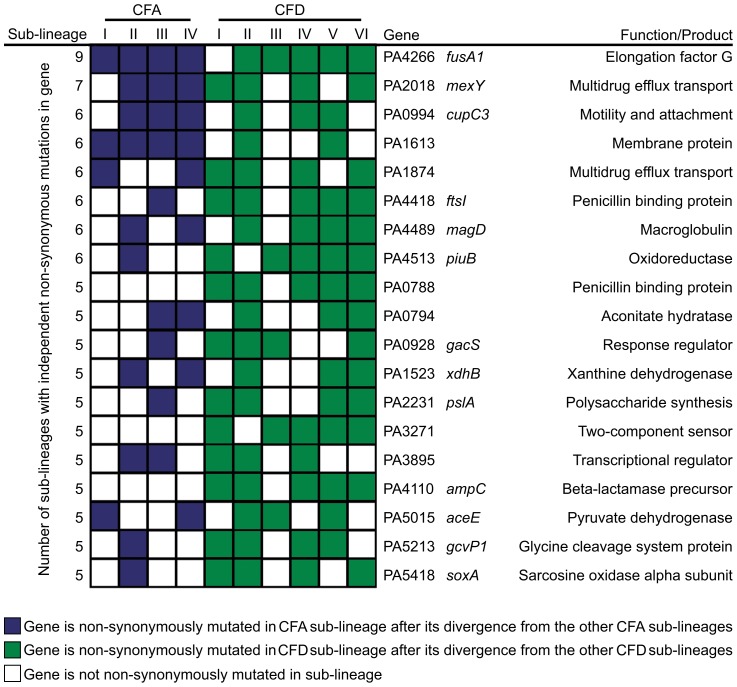
Pathoadaptive genes convergently mutated in CFA and CFD sub-lineages. The analysis was performed based only on non-synonymous mutated genes that were altered independently in at least half of the 10 evolving sub-lineages CFA I–IV and CFD I–VI.

However, it is conceivable that positively selected genes remain “hidden” among the much larger number of genes that have accumulated mutations by genetic drift. Evolving populations of *P. aeruginosa* presumably accumulate adaptive mutations in response to the human host environment in which they propagate. We would therefore expect to observe parallelism in the adaptive genetic routes of the different lineages. To confirm such convergent evolution, we attempted to identify genes that underwent parallel mutation in the 10 sub-lineages (four CFA sub-lineages and six CFD sub-lineages) that coexisted over many years (Figure 2). We analyzed our dataset by selecting those genes that were independently mutated in at least half of the parallel evolving sub-lineages (see Materials and Methods). Forty genes were found to be frequently mutated across the sub-lineages (Table 2), suggesting that the parallel mutation of these genes was due to positive selection for mutations. Consistently, the signature for selection for SNPs accumulated in the 40 genes (dN/dS = 0.97) was significantly higher than the ratio obtained for SNPs affecting all other genes (dN/dS = 0.75; *P* = 0.029 by Fisher's exact test), suggesting that these mutations were positively selected during evolution. Analysis of the 40 genes was further focused on those that were non-synonymously mutated in at least half of the 10 sub-lineages (Figure 4). Several of these genes were associated with functions related to CF host adaptation. In particular, *ftsI*, *ampC*, *fusA1*, *mexY*, PA1874, and PA0788 are involved in resistance to antibiotics commonly used in CF therapies, *i.e.*, betalactams, aminoglycoside, quinolones, chloramphenicol, trimethoprim, and imipenem [29], [47], [48], [49], [50], [51], [52], [53]. Even though the GacA/GacS system is required for activation of genes involved in chronic persistence, *gacS* mutants are prone to generate stable and stress-tolerant small colony variants (SCVs) when growing in biofilms, exposed to stress factors [54], [55], or *in vivo* [56], suggesting that the absence of GacS may confer some additional advantage for persistence in the CF lung. *fusA1* encodes the elongation factor EF-G1A, which confers resistance to the antibiotic argyrin in *P. aeruginosa* [57], [58]. Chung *et al.* recently reported independent *fusA1* mutations in two CF patients and suggested that these mutations are involved in regulation of virulence through a ppGpp-dependent stringent response [43]. On the other hand, the gene *pslA* is involved in biofilm formation [59], and *cupC3* is associated with motility/attachment [60]. Lack of motility is a trait frequently observed in isolates from chronically colonized patients, and may give *P. aeruginosa* a survival advantage in chronic CF infection by enabling it to resist phagocytosis and conserve energy [61].

**Table 2:** Evidence for parallel evolution. Genes identified as being independently mutated in at least a half of the coexisting CFA I–IV and CFD I–VI sub-lineages.

Gene ID	Gene name	Function classification^a^	Total number of independent mutations	Number of sub-lineages convergently mutated	
				CFA	CFD
PA3327		Adaptation, Protection	5	2	3
PA4110	*ampC*		6	1	5
PA0074	*ppkA*	Adaptation, Protection; Translation, post-translational modification, degradation; Protein secretion/export apparatus	5	2	3
PA5015	*aceE*	Amino acid biosynthesis and metabolism; Energy metabolism	5	2	3
PA5418	*soxA*	Carbon compound catabolism	5	1	4
PA4418	*ftsI*	Cell division; Cell wall/LPS/capsule	6	1	5
PA2231	*pslA*	Cell wall/LPS/capsule	5	1	4
PA2238	*pslH*		5	1	4
PA5213	*gcvP1*	Central intermediary metabolism; Amino acid biosynthesis and metabolism	5	1	4
PA4285	*recC*	DNA replication, recombination, modification and repair	5	2	3
PA0794		Energy metabolism	5	2	3
PA1613		Membrane proteins	6	4	2
PA2072			5	3	2
PA3234		Membrane proteins; Transport of small molecules	5	2	3
PA3920			5	1	4
PA4719			5	3	2
PA0994	*cupC3*	Motility & Attachment	6	3	3
PA1099	*fleR*	Motility & Attachment; Transcriptional regulators; Two-component regulatory systems	5	1	4
PA1523	*xdhB*	Nucleotide biosynthesis and metabolism	5	2	3
PA3763	*purL*		7	4	3
PA3895		Transcriptional regulators	5	2	3
PA4937	*rnr*	Transcription, RNA processing and degradation	5	2	3
PA4266	*fusA1*	Translation, post-translational modification, degradation	9	4	5
PA2018	*mexY*	Transport of small molecules; Membrane proteins; Antibiotic resistance and susceptibility	7	3	4
PA0928	*gacS*	Two-component regulatory systems	6	1	5
PA1336			5	2	3
PA3271			6	0	6
PA2378		Putative enzymes	6	2	4
PA4513			6	1	5
PA4489	*magD*	Hypothetical, unclassified, unknown; Adaptation, protection	6	2	4
PA1874		Hypothetical, unclassified, unknown; Antibiotic resistance and susceptibility	8	3	5
PA1669		Hypothetical, unclassified, unknown; Membrane proteins	5	1	4
PA0454		Hypothetical, unclassified, unknown	6	2	4
PA0788			5	0	5
PA2077			5	1	4
PA2151			5	2	3
PA2635			5	1	4
PA3728			5	2	3
PA4735			5	3	2
PA4836			5	1	4

a The categories used for functional classification were as described in the The *Pseudomonas aeruginosa* Community Annotation Project (http://www.pseudomonas.com).

Alterations in several genes related to bacterial catabolism (*e.g.*, *aceE*, *gcvP1*, *soxA*, *xdhB*, PA0794) were also observed, suggesting that the inactivation of certain metabolic functions may be a common trait related to CF host adaptation (see below).

The concurrent alteration of specific genes or functions related to adaptation to the CF airway environment provides strong evidence for parallel evolution not only across CFA and CFD lineages, but also across intra-patient coexisting sub-lineages. Certain genes were convergently but exclusively mutated among CFD sub-lineages, *e.g.*, *ampC* (beta-lactamase precursor), PA0788 (penicillin binding protein), and PA3271 (two-component sensor). These findings suggest the occurrence of in-host parallel evolutionary processes resulting from specific selective pressures from differential antibiotic treatments.

Mutations in the global regulators *mucA*, *algT*, *rpoN*, and *lasR* are related primarily to adaptation to the CF airway environment [10], [17], [33], [62], [63], [64]. However, our analyses did not reveal such mutations because they arose in the ancestral isolates before diversification into sub-lineages (Table S4). The entire population from CFA had a mutation in *mucA*, whereas the population from CFD had mutations in *lasR* and *rpoN* (Table S4). These findings indicate that mutations in these regulator genes were specifically fixed in the respective bacterial populations during early in-host evolution.

### Accumulation of mutations in MRS genes and the emergence of a subpopulation with reduced mutation rate

#### MRS genes in the CFA panel

To investigate the molecular basis for the strong mutator phenotypes observed in CFA_2010 and CFD_2011 clones, we analyzed the sequences of the MRS genes *mutS* and *mutL* from the whole-genome sequence data and looked for potential alterations.

Mutations in both MutS (T247P) and MutL (H469R) proteins were found in the ancestral isolate CFA_2004/01 (Table 1). This same combination of mutations (termed allelic combination SL1) was also present in the later isolate CFA_2007/01 and in seven of the 11 CFA_2010 clones. The remaining four CFA_2010 isolates showed two additional missense mutations in MutS (L794P and W160R) and one in MutL (L389S). These two new MRS allelic combinations were termed SL2 and SL3, respectively (Table 1). SL1, SL2, and SL3 were distributed in different phylogenetic clusters (Clusters I, IVa, and IVb for SL1, Cluster II for SL2, Cluster III for SL3) (Table 1 and Figure 2A), demonstrating the coexistence of multiple polymorphisms in the MRS genes as a reflection of the observed genomic diversity.

We further sequenced the *mutS* and *mutL* genes in 19 additional CFA_2010 isolates to extend our analysis to 30% of the population (30 isolates). Fourteen of these newly sequenced isolates carried the ancestral SL1 combination, five carried SL3, and none carried SL2, leading to final prevalences of 70% for SL1, 3% for SL2, and 27% for SL3. These observations suggest that SL1 was predominant and persisted over time among the within-patient coexisting isolates.

The logical next question was whether the different mutations observed in *mutS* and *mutL* in CFA_2010 isolates were able to alter the function of their respective gene products, resulting in the mutator phenotype. To address this point, we attempted to restore a wild-type normo-mutable phenotype from SL1, SL2, or SL3 mutator strains by complementation with a plasmid carrying the wild-type *mutS* or *mutL* gene (Materials and Methods). Our results indicated that the ancestral mutation in MutL (H469R), but not those in MutS, was responsible for the mutator phenotype observed in isolates that carried not only the SL1 combination but also SL2 and SL3 (Text S1). Although *mutS* has been shown to be the main target for acquisition of a stable hypermutor phenotype [15], [65], mutations in *mutL* are a frequent cause of MRS deficiency in *P. aeruginosa* CF isolates [17], [66].

#### MRS genes in the CFD panel

Analysis of the CFD mutator collection revealed that every CFD_2011 isolate, whether mutator or normo-mutator, harbored the same ancestral frameshift mutation in *mutS* (−CG_1551_) (Table 1). The normo-mutable isolates (grouped in Cluster IV) also harbored a +CC_334_ insertion (Table 1) in a 5-bp G∶C simple sequence repeat (SSR) of *mutS*. This insertion generates a premature stop codon at position 347 bp from the ATG start codon. In addition, the +CC334 insertion produced an ATG codon at 1822 bp, resulting in restoration of the frameshift now coding for a C-terminal peptide of 248 amino acids, which correspond to MutS functionally essential domains [67], [68] (File S1). This finding suggested that the +CC_334_ insertion is associated with the reduced mutation frequency in normo-mutators. To test this concept, we cloned the *mutS* ^+CC334−CG1551^ allele into the pMC5 plasmid (Table S5) and performed complementation assays in *mutS*-deficient strain PAO1 (Materials and Methods). Nonetheless, the above allele did not revert the mutator phenotype, and we found no extra copies of a functional *mutS* allele in the normo-mutable genomes.

We next examined the mutational spectra of CFD_2011 isolates in Cluster IV. The proportion of transversions showed a significant increase (*P*≤0.05 in two-tailed T-test adjusted by Bonferroni correction), from 5.8% in contemporary mutators belonging to Cluster V and VI (with the ancestral *mutS* ^−CG1551^ allele) to 10.6% in normo-mutators grouped in Cluster IV (with the *mutS* ^+CC334−CG1551^ allele) (Figure S2).

We sequenced the *mutS* gene in 19 additional CFD_2011 mutator isolates (similarly to the studies of the CFA population) and found that all of them (100%) carried the −CG_1551_ mutation in *mutS*, indicating that the proportion of clones with reduced mutation frequency remains low in the CFD population (∼6%).

### Mutational snapshot of SSRs in *P. aeruginosa* mutator populations

Our previous *in vitro* studies showed that, in a MRS-deficient background, G∶C SSRs constitute hotspots capable of biasing mutagenesis toward a specific genetic pathway [34], [38]. Our recent studies of *P. aeruginosa* PACS2 and epidemic DK2 strains demonstrated the same phenomenon at a genome-wide level in CF *in vivo* chronic airway infection [18], [39].

The present study design allows examination of such a genome-wide effect of biased mutagenesis in large populations obtained at single time points, and observation of SSR instability in coexisting isolates.

**Figure 5:**
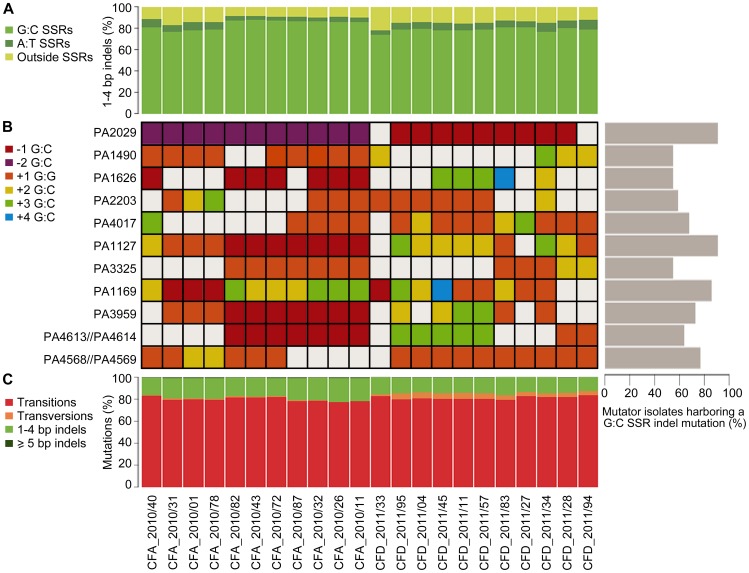
Mutational spectra and top mutated homopolymeric G∶C SSRs in CFA_2010 and CFD_2011 contemporary isolates. (A) Percentage of 1–4 bp insertions/deletions located in G∶C and A∶T homopolymeric sequences. (B) The heat map represents individual indels mutations in homopolymeric G∶C SSRs of ≥6 bp, which were mutated in at least half of the coexisting isolates in both CFA and CFD lineages. The color-code indicates the type of mutation. *Right*: Percentage of MRS-deficient isolates harboring a indel mutation in each analyzed G∶C SSR. (C) Mutations in CFA_2010 and CFD_2011 isolates were analyzed based on the percentage of transitions, transversions, and insertions/deletions.

Analysis of types of mutations occurring in both the CFA_2010 and CFD_2011 isolates revealed a mutational spectrum typical of MRS-deficient strains. Transitions (∼80%) and small indels (1–4 bp) (∼16%) were the most frequently observed mutations in both collections (Figure 5C). The most prevalent transition was G∶C→A∶T, accounting for 65% and 62% of total transitions in CFA and CFD lineages, respectively. Of the 1–4 bp indels,>80% were located within a homopolymeric SSR, and ∼75% were located specifically in G∶C SSRs (Figure 5A). In contrast, indels in A∶T SSRs accounted in average for 5.2% and 6.7% of the 1–4 bp indels in CFA and CFD isolates, respectively (Figure 5A).

Based on this strong skewing of MRS spectra toward small indels in G∶C SSRs, we selected homopolymeric G∶C SSRs of ≥6 bp, which were mutated in at least half of the coexisting isolates in both CFA and CFD lineages, and analyzed their mutational dynamics at the intra-population level. Eleven of these highly mutated G∶C SSRs harbored 2–5 distinct indel mutations accounting for independent mutational events (Figure 5B). A single SSR was observed to be either unaltered or modified by different indel mutations even in coexisting isolates from the same cluster. Analysis of these 11 G∶C SSRs in the normo-mutable CFD_1991/01 isolate showed no mutations. Using genome data available online, we evaluated the occurrence of indel mutations in these G∶C SSRs in 12 normo-mutable *P. aeruginosa* strains (PAO1, PA14, M18, NCGM2.S1, B136-33, RP73, 39016, PACSC2, 2192, C3719, DK2, LESB58; the latter five are normo-mutable isolates obtained from CF infections), whose genomes have been sequenced and are available in the *Pseudomonas* Genome Database (www.pseudomonas.com) [69]. Our survey revealed that these 12 strains harbored no indel mutations in the analyzed G∶C SSRs, even though large G∶C SSRs are considered to be “hotspots” for mutagenesis.

One of the identified homopolymers is located in a gene (PA4071/PADK2_03970) which has been previously suggested to be preferentially mutated in mutators and to represent a mutator-specific target of adaptive mutations [18].

These findings suggest a scenario in which MRS-deficient populations generates a vast of genetic diversity due to G∶C SSR instability. In this scenario, genes containing large G∶C SSRs constitute continual sources of genetic diversification primarily in mutator bacterial populations.

### Global analysis of the catabolic capacities of CFA and CFD mutator lineages during long-term evolution in CF infections

**Figure 6:**
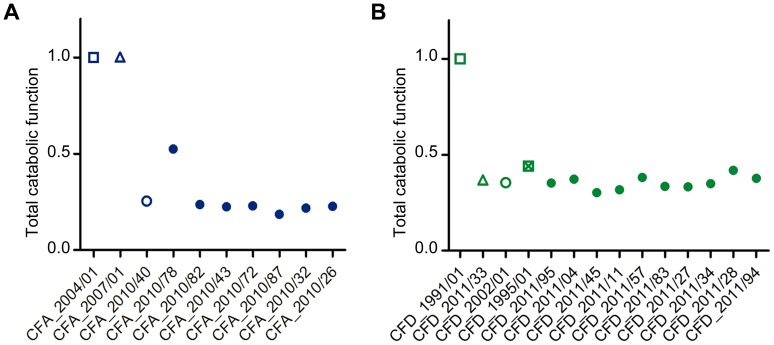
Average total catabolic function of isolates from CFA and CFD lineages. Total catabolic function was calculated relative to CFA_2004/01 and CFD_1991/01 as a weighted average across all substrates for each CFA (A) and CFD (B) isolate. Total catabolic function was defined as 1 for the reference levels (CFA_2004/01 and CFD_1991/01). Lower values indicate decay. Isolates CFA_2010/01, CFA_2010/11, and CFA_2010/31 were excluded from the analysis because significant dispersion was observed in the duplicates.

We evaluated the dynamics of phenotypic changes in the 27 *P. aeruginosa* CFA and CFD isolates by determining global catabolic activities (the “catabolome”). Biolog phenotype microarrays were used to monitor the catabolic profiles of each isolate with various C and N sources (Table S7). The total catabolic functions in the isolates were greatly reduced (average reduction 73.5% and 63.8%, respectively) in comparison with those of the CFA_2004/01 and CFD_1991/01 ancestors (Figure 6). This extensive loss of functions led to homogeneous populations in both the CFA and CFD lineages, with slight catabolome variation among clones. Catabolic function reduction thus appears to be a phenotypic pattern shared by CFA and CFD mutator lineages. Accordingly, genes related to catabolism were convergently mutated in both the CFA and CFD lineages (Figure 4). This phenomenon may be partially responsible for the decreased catabolic phenotype.

## Discussion

This study provides a complete panorama of the genomic diversity that shapes the structure of *P. aeruginosa* mutator populations during long-term adaptation to the CF airway environment. We combined a longitudinal study with an extensive cross-sectional approach, including multiple isolates obtained from single sputum samples, which allowed in-depth analysis of population diversity (Figure 1). We utilized *P. aeruginosa* panel collections from two chronically infected CF patients, CFA (Argentinian) and CFD (Danish), with time spans of 6 yrs and 20 yrs, respectively, from initial to later stages of chronic infection. Our comprehensive study design included whole-genome sequencing and high-throughput phenotypic approaches, calculation of mutation frequencies, phylogenetic estimation of time points of sub-lineage diversification, and analysis of *mutS* and *mutL* genes to obtain a wide-ranging depiction of hypermutability in CF.

We expected (and confirmed) that each of the two patients was infected by a single non-epidemic *P. aeruginosa* clone that did not present, during the initial stages of infection, the pathoadaptive mutations displayed by epidemic clones [42], [70], [71], [72]. We were therefore confident that our analysis addressed specific and independent in-host evolutionary processes. Mutator strains were highly prevalent in both patients, essentially dominating the populations. The proportion of mutators was ≥90% in the single time point 90-isolate collections, indicating that mutators, once selected, dominated the CFA and CFD infecting populations. The observed prevalence of within-patient mutators was much higher than the values reported in previous studies [8], [13]. These findings indicate that although *P. aeruginosa* may persist throughout the course of chronic infection without ever acquiring the mutator phenotype, mutator strains may become prevalent and even dominate the whole population under certain yet-unknown conditions. This concept is supported by the observation that two patients of different ages from geographically distant locations, infected with different non-epidemic *P. aeruginosa* clones and subjected to different therapeutic protocols, underwent overlapping evolutionary trajectories that led to complete domination of mutators.

Recent reports have demonstrated high diversity at the phenotypic level among *P. aeruginosa* populations from CF lung infections [8], [9], [73]. However, there have been no genome-wide studies of such diversity in bacterial populations from the same clinical samples. The global picture of genetic structure of intra-patient mutator populations in the present study reveals significant genomic diversity driven by high accumulation of mutations (Figures 2 and 3), reflected by the typical MRS spectra (Figure 5). The distribution and combination of thousands of mutations result in a unique genotype for every isolate, allowing long-term persistence in the CF airway environment. The observed genomic variation into the CFA and CFD lineages indicates that the population structure in each case was not determined by homogeneous single dominating clones, but occurred through multiple evolutionary genetic pathways that adapted equally to the CF airway environment and allowed the coexistence of diverse subpopulations for many years. We determined that this high genomic diversity, to an equal degree in the two patients, spread out from the establishment of chronic infection. Interestingly, MRS genes were also characterized by the coexistence of multiple polymorphisms. However, underlying the polymorphisms within the MRS genes, there is an ancestral mutation that was fixed in each CFA and CFD population and is apparently responsible for hypermutability. These findings suggest the existence of common selective forces acting on MRS inactivation in the two patients.

The long-term evolution of the *P. aeruginosa* CFA and CFD lineages was signed mainly by purifying selection and/or genetic drift. There are conflicting reports regarding whether genomic evolution of CF isolates shows signatures of positive selection [10] or (in contrast) genetic drift and/or purifying selection [32]. Our results strongly support the latter concept; *i.e.*, that genomic signatures of purifying selection and/or genetic drift are not inherent consequences of mutators, but are characteristic of the genetic adaptation processes underlying *P. aeruginosa* persistence in chronic lung infections.

According to our observations, the large number of mutations were for the most part distributed randomly among the *P. aeruginosa* mutator genome (Figure 3). However, we also identified a group of genes that were convergently mutated in multiple genomes by independent mutational events (Table 2 and Figure 4). Most of these genes code for functions related to pathogenicity (*e.g.*, antibiotic resistance, virulence, motility, attachment), suggesting that they were positively selected as beneficial mutations. We note that five of the genes (*ampC*, *ftsI*, *fusA1*, PA3271, PA2018) were also found to be mutated in isolates obtained from the epidemic DK2 clone [18], providing evidence of parallel evolution for certain specific traits among different *P. aeruginosa* lineages.

As we have reported recently for the PACS2 [39] and epidemic DK2 *P. aeruginosa* strains [18], the impact of hypermutability on the evolution of the CFA and CFD lineages is reflected by the high tendency of G∶C SSR-containing sequences to be mutated (Figure 5A). This finding confirms that genes which maintain G∶C SSRs in their coding region and/or in neighboring regulatory sequences are highly unstable in an MRS-deficient background and may be mutator-specific targets of adaptive mutations. This concept is extended here by the demonstration that large G∶C SSRs, as DNA sequences *per se*, are highly polymorphic in single time point populations, indicating that they are continual sources for diversification (Figure 5B). This SSR-driven diversity is not observed in genomes of other *P. aeruginosa* strains, even of normo-mutable CF clones. Our present and previous results [18], [34], [38], [39], taken together, demonstrate a clear association between MRS-deficiency and G∶C SSR instability, which exerts a global effect along the entire genome. The impact of hypermutability during evolution of *P. aeruginosa* in the CF airway environment is not simply a major, rapid acquisition of mutations in quantitative terms. In contrast with SNPs, indels that are not multiples of three produce frameshifts in the coding sequence of genes and thereby affect gene function. Indels in G∶C SSRs may play an important role in the evolutionary process and in relation to mutator competitiveness. Along this line, nine of the 11 analyzed G∶C SSRs (Figure 5B) were located in coding regions of the genome. Five of these genes are predicted to encode for hypothetical proteins with no assigned function. On the other hand, some G∶C SSRs were positioned in genes functionally related to transcriptional regulators (PA1490), adaptation-protection (PA1127), membrane proteins, and transport of small molecules (PA1626, PA2203).

A small percentage (6%) of the CFD population has a reduced mutation frequency similar to that of normo-mutable strains. This subpopulation, which is grouped in a single cluster (Cluster IV in Figure 2), had the highest accumulation of mutations observed in the whole CFD collection (Table S3). This observation posed the question whether the mutational load of these clones is too heavy to continue supporting a mutator phenotype. However, the normo-mutable subpopulation carried the same *mutS* loss-of-function mutation (Table 1) as the mutator isolates. Phylogenetic analysis indicated that isolates from Cluster IV had arisen from a mutator subpopulation at some undetermined point in branch J (Figure 2). Our sequencing data suggested that the most feasible explanation is the emergence of secondary mutations, in genes not belonging to the MRS, that compensate for *mutS* hypermutability, since neither reversion of the original −CG_1551_ nor duplication of the *mutS* gene was observed in normo-mutable genomes. Although the new +CC_334_ mutation restored in part the *mutS* reading frame, this fact did not account for the reduction in mutation frequency observed in these clones. In a previous study, *E. coli* MRS-deficient populations *in vitro* evolved a compensation of the mutator phenotype based on secondary mutations in genes related to oxidative stress response which, although selected for different increasing-fitness traits, resulted in a reduction of the mutation rate [74]. Future studies will elucidate the mechanisms underlying the observed reduction in mutation frequency of these *mutS*-deficient *P. aeruginosa* strains.

How are these mutator populations maintained over extended periods of time, and even able to accumulate greater numbers of mutations? The chronically infected CF lung is a uniquely challenging habitat in which *P. aeruginosa* must cope with aggressive immune system responses, intense antibiotic therapies, and/or competition with other resident microorganisms. This environment presents stressful and variable conditions, and multiple mutations may occur that increase fitness. On the other hand, favorable nutritional conditions of CF airways [75], [76] may sustain the inactivation of certain functions that are no longer necessary for survival, particularly in this environment. In this regard, CF isolates have been reported to accumulate a high number of auxotrophies – higher than in other studied environments [77]. The results obtained here for CFA and CFD catabolomes indicate overall reduction of total catabolic activities (Figure 6). Reduction of catabolic functions appears to be part of a more general adaptive process of *P. aeruginosa* residing for long periods in the CF lung, because such reduction has also been observed in normo-mutable strains [42]. The large genome of *P. aeruginosa* may have the potential to undergo reductive evolution, with elimination of functions that are redundant and/or dispensable in the CF host environment, thereby buffering the heavy mutational load observed [78].

Based on these considerations, we hypothesize that, during long-term evolution of *P. aeruginosa* in CF airways, the increased availability of certain beneficial mutations, in combination with a whole-genomic signature of neutral evolution, provided favorable conditions to increase the percentage of mutators, until reaching a frequency at which mutators dominated both the CFA and CFD populations. *P. aeruginosa* adaptation to the CF lung is presumably manifested through selection of multiple genetic combinations [79], and hypermutability is consequently maintained over time as a constant source of genetic variation.

## Materials and Methods

### *P. aeruginosa* isolate collections

Clinical *P. aeruginosa* isolates were obtained from sputum samples from two CF patients at the Hospital de Niños Santísima Trinidad (Córdoba, Argentina) (patient CFA) and the Copenhagen CF Centre at Rigshospitalet (Copenhagen, Denmark) (patient CFD). Patient age at the time of the first isolate collection was 8 and 23 yrs, respectively. The onset of chronic infection with *P. aeruginosa* was 2001 and 1986, respectively. Chronic pulmonary infection was defined as the persistence of *P. aeruginosa* in sputum for 6 consecutive months, or less if the persistence was combined with presence of two or more precipitating antibodies against *P. aeruginosa* [33]. The criteria for choosing these patients were: (i) chronic airway infection by *P. aeruginosa*; (ii) absence of transmissible *P. aeruginosa* clones; (iii) presence of single clonal *P. aeruginosa* infecting lineages throughout the course of infection; (iv) appearance of MRS-deficient *P. aeruginosa* mutators during the course of infection.

Sputum samples were collected by expectoration during routine hospital visits, stored on ice, and processed immediately. Sputa were liquefied by addition of an equal volume of Sputolysin (Calbiochem), diluted, and plated onto *Pseudomonas* isolation agar (BD Biosciences), which promotes growth of *P. aeruginosa* and other *Pseudomonas* species. For cross-sectional analysis, 90 isolates were taken randomly from a single sputum sample. Isolates were maintained and stored at −70°C in glycerol stock solution. For assays, isolates were subcultured from the frozen stocks onto Luria Bertani (LB) agar plates, and inocula were prepared from overnight cultures in LB broth at 37°C with appropriate aeration.

### Genotyping analysis

Genotyping analysis of the *P. aeruginosa* isolates was performed by Randomly Amplified Polymorphic DNA (RAPD), using primer 272 (Table S5) [5], [80]. Banding patterns from electrophoretic gels were analyzed by GelPro Analyzer V. 6.3 software. Epidemiological relatedness was evaluated by pulsed-field gel electrophoresis (PFGE) using *SpeI* enzyme as described previously [81]. DNA macrorestriction patterns were interpreted according to the criteria of Tenover et al. [82]; *i.e.*, isolates with PFGE patterns differing by (i) 1–3 bands are closely related clones; (ii) 4–6 bands are possibly related clones; (iii) ≥7 bands belong to different strains. Clonal relatedness among the isolates selected for genome sequencing analysis was evaluated by single-nucleotide polymorphism (SNP) typing using AT biochips (Clondiag Chip Technologies, Germany) [83]. Strain assignment was performed by visual array analysis using a hexadecimal code as described previously [45].

### Mutation frequency measurement

Mutation frequencies were estimated as described previously [13], [15]. In brief, five independent colonies of each isolate were grown overnight in 10 ml LB medium at 37°C with appropriate aeration. Cultures were collected by centrifugation at 3000 rpm for 10 min and resuspended in 1 ml LB medium. Serial ten-fold dilutions were plated on LB agar with and without 300 µg/ml rifampicin. After 36 h incubation (48 h for slow-growing strains) at 37°C, colonies were counted and the mean percentage of mutants was estimated. Strains were considered hypermutable if the mutation frequency was at least 20-fold higher than that of control strain PAO1 [13]. Frequencies were determined from two independent experiments.

### Genome sequencing

Genomic libraries were prepared as described previously [42] and sequenced on an Illumina Hiseq2000 platform, generating 100 base paired-end reads using a multiplexed protocol. A total of 224 million paired-end reads were generated to yield average genomic coverage of 33–207 fold (all average coverage depths were ≥82× except for CFD287_2011/94 (33×) and CFD_2011/27 (52×)). Genome sequence reads were deposited in the European Nucleotide Archive (ENA/SRA ERP002379).

Illumina reads from isolates CFA_2004/01 and CFD_1991/01 were *de novo* assembled using Velvet software V. 1.2.07 [84] using the following settings: -ins_length 238 and 244 for CFA_2004/01 and CFD_1991/01, respectively; -ins_length_sd 54.4 and 50.3 for CFA_2004/01 and CFD_1991/01, respectively; -exp_cov 291.1 and 296.7 for CFA_2004/01 and CFD_1991/01, respectively; -cov_cutoff 10; -min_contig_lgth 500. Selected kmer sizes were 51 and 39 for CFA_2004/01 and CFD_1991/01, respectively. Each *de novo* assembly was used as a reference genome sequence to map reads from the remaining CFA and CFD genome sequences using Novoalign V. 2.08.02 (Novocraft Technologies) [85]. Pileups of the mapped reads were processed by SAMtools V. 0.1.7 [86]. SNPs were identified by the varFilter algorithm in SAMtools (samtools.pl varFilter -d 3 -D 10000), and only unambiguous SNPs with quality scores (Phred-scaled probability of sample reads used as homozygous reference) of ≥50 (*i.e.*, *P*≤10^−5^) were retained. Read alignments surrounding all putative indels were realigned using GATK V. 1.0.5083 [87], and microindels were extracted from the read pileup using the following criteria: (i) quality score ≥500; (ii) root-mean-square (RMS) mapping quality ≥25; (iii) support by ≥20% of the covering reads. The false-negative rates obtained were 2% and 3% by *in silico* introduction of random base-substitutions and microindels (lengths 1–10 bp), respectively. All sites with putative polymorphisms in the pileup of reads from the reference were excluded to avoid false-positives resulting from errors in the reference assembly or general mapping errors. MUMmer3 [88] was used for whole-genome alignments. Mutations were described according to their relative gene positions in orthologs of *P. aeruginosa* reference strains PAO1, PA14, and LESB58 with completed genome sequences [69]. If no ortholog was found in the reference strains, the mutation was described by the GenBank ID of the homolog sequence (Genbank_ID:ORF_name: Position) or as “Not annotated”.

Phylogenetic trees based on the SNP mutations identified in each alignment were constructed using maximum-parsimony analysis as described previously [42].

For calculation of selection coefficients (dN/dS ratio), we assumed that codon usages were identical to those in strain PAO1, in which 25% of random mutations are synonymous [42], and that the probability of the observed number of nonsynonymous SNPs, given the expected number of SNPs, is calculated from the Poisson distribution.

### Bayesian evolutionary analysis

Bayesian analysis of evolutionary rates was performed using BEAST V. 1.7.2 [46]. The BEAST program was run with the following user-determined settings: a lognormal relaxed molecular clock model, which allows rates of evolution to vary among the branches of the tree, and a HKY substitution model, which distinguishes between the rate of transitions and transversions and allows unequal base frequencies. Mutation rates were calculated from chains of 100 million steps, sampled every 5,000 steps. The first 10 million steps of each chain were discarded as a burn-in.

### Identification of genes subject to convergent evolution

To identify genes subject to convergent evolution, we picked out those that were mutated in at least half of the in parallel evolving sub-lineages CFA I–IV and CFD I–VI. The sub-lineages/clusters were defined as follows (see Figure 2). CFA lineage: Cluster I: branches Z, Y; Cluster II: branch A; Cluster III: branches C, D, E, F, G; Cluster IV: branches N, J, T, U, R, S, O, Q, K, I, M. CFD lineage: Cluster I: branch A2; Cluster II: branch C; Cluster III: branch E; Cluster IV: branches J, I, L, M, N, K, P, O; Cluster V: branches U, T, W, V, X; Cluster VI: branches R, G, Q. For example, gene *aceE* was hit by independent mutations in CFA branch J (sub-lineage CFA-IV), CFA branch Y (sub-lineage CFA-I), CFD branch C (sub-lineage CFD-II), CFD branch E (sub-lineage CFD-III), and CFD branch T (sub-lineage CFD-V). To rule out the possibility that high accumulation of mutations resulted from particularly large gene sizes, mutations from large genes (≥10 kb) were excluded from the analysis.

### Minimum spanning trees (MSTs)

MSTs for the set of mutated genes within each CFA and CFD lineage were obtained using Prim's algorithm (Prim, 1957) as implemented in the Info-Gen program [89]. In this model, nodes represent an isolate's set of mutated genes, the distance between a pair of nodes is shown as the number of distinctive mutated genes, and nodes are connected in such a way that the sum of the distances is minimized (Table S6).The network connects each genotype to all other genotypes through a pathway of mutated genes. The CFA_2004/01 and CFD_1991/01 genomes were used as starting points of the network of the corresponding MSTs for each lineage. The number of mutated genes along the network was used as a measure of divergence between two given genotypes.

### Sequence analysis of the *mutS* and *mutL* genes

Genomic DNA of *P. aeruginosa* CF isolates was extracted using a DNA Isolation Kit (Qiagen). Primers used for PCR amplification and DNA sequencing are listed in Table S5. PCR amplifications were performed with the following conditions: 8 min at 95°C, 33 cycles of 1 min at 94°C, 1 min 20 sec at 60°C, 2 min at 72°C, and a final extension of 10 min at 72°C. PCR products were cleaned with a Gel Purification Kit (Qiagen), and both strands were sequenced directly using the same PCR primers (DNA Sequencing Facility, Univ. of Chicago, IL, USA). To score mutations within the gene, sequencing results were compared with the corresponding gene sequence of strain PAO1 (www.pseudomonas.com) using the BLAST program of the NCBI database (www.ncbi.nlm. nih.gov/blast/).

### Construction of plasmids pMC5-MutS−CG_1551_ and pMC5-MutS+CC_344_−CG_1551_

Two constructs of plasmid pMC5-MutS [90], which contains the full coding region of the *mutS* gene from strain PAO1, were generated by introducing −CG_1551_ and +CC_334_−CG_1551_ mutations to produce plasmids pMC5-MutS−CG_1551_ and pMC5-MutS+CC_344_−CG_1551_, respectively. To introduce these mutations, the *mutS* genes from CFD_2011/27 and CFD_2011/11 isolates were amplified by PCR using oligonucleotides *mutS*-for1 and *mutS*-rev4 (Table S5), ligated to the pGEM-T Easy vector (Promega), and cloned in the *Nde*I/*Eco*RI restriction sites of pMC5-*mutS*, in which the *mutS* gene has been digested with *Nde*I/*Eco*RI. Both plasmids were propagated on *E. coli* DH5α (Invitrogen) transformed by heat shock by standard procedures.

### Complementation assays with pMC5-MutS, pMC5-MutL, pMC5-MutS−CG_1551_, and pMC5-MutS+CC_344_−CG_1551_ plasmids

To evaluate the genetic basis of the mutator phenotype, plasmids pMC5-*mutS* [90] and pMC5-*mutL* [91] were successively transferred into the mutator CFA and CFD isolates. To analyze +CC_344_−CG_1551_ mutations in the *mutS* gene, plasmids pMCS-MutS−CG_1551_ and pMCS-MutS+CC_344_−CG_1551_ were independently transferred into reference strain MPAO1MS. All plasmids were transferred by electroporation as described by Choi and Schweizer [92], and transformed strains were selected on LB agar plates supplemented with 100 mg/ml gentamicin. Complementation was checked by the rifampin test described above. For each strain, complementation was confirmed for three independent transformed colonies.

### Catabolic analysis by Biolog Phenotype MicroArrays

Phenotype MicroArrays PM1 and PM3B (Biolog; Hayward, CA, USA) were performed according to the manufacturer's instructions [93], [94]. Export of Omnilog data was performed using Omnilog OL-FM/Kin software V. 1.20.02 (Biolog). Phenotypes were determined based on the parameter “ave area” (the area beneath the respiration curve of reduced tetrazolium vs. time). For comparison of clinical isolates, data were exported after 72 h incubation. Data analysis and statistical analysis were performed using R Project V. 2.10.0 (http://www.R-project.org). The R packages used for analysis were “bioDist” (B. Ding, R. Gentleman, V. Carey). Total catabolic function was calculated as described previously [95].

### Statistical analyses

Statistical analyses were performed using a two-tailed T-test adjusted by Bonferroni correction. Differences with *P*≤0.05 were considered statistically significant.

### Ethics statements

The *P. aeruginosa* isolates were obtained from sputum samples from one CF patient at the Hospital de Niños Santísima Trinidad (Córdoba, Argentina) (patient CFA) and one CF patient at the Copenhagen CF Centre at Rigshospitalet (Copenhagen, Denmark) (patient CFD), as byproducts of the routine established for bacterial typing and antimicrobial susceptibility testing. *I.e.*, sputum sampling was not performed for the purposes or intent of the present study; isolates recovered from these sputa were simply derivatives of routine CF patient therapeutic controls. The therapeutic treatments of the two patients were not modified in any way as a consequence of the results obtained in this study. The research protocols followed in this study were approved and reviewed by the Ethics Committee of the Hospital de Niños Santísima Trinidad, Córdoba, Argentina and the local ethics committee, Region Hovedstaden, Copenhagen, Denmark (H-A-141 and H-1-2013-032). Both of the patients gave informed consent.

## Supporting Information

Figure S1 SNP mutations in CFA and CFD lineages. SNPs were analyzed in terms of percentages of nonsense, missense, silent, intergenic, and non-annotated SNPs.
(TIF)

Figure S2 Mutational spectra of CFD normo-mutable and mutator genomes. SNPs were analyzed in terms of the percentages of transitions and transversions in Clusters IV (normo-mutators) and V–VI (mutators). The total number of SNPs from CFD maximum-parsimony phylogenetic tree branches J-I-L-P-O-N-K-M and S-U-T-W-V-X-Q-R-G were considered for Clusters IV and V–VI, respectively (see Figure 2). *: significant difference (P<0.05).
(TIF)

Table S1 *De novo* assemblies of *P. aeruginosa* CFA_2004/01 and CFD_1991/01 genomes.
(DOC)

Table S2 Illumina sequencing and mapping statistics.
(DOC)

Table S3 Numbers of total SNPs and indels in the sequenced genomes.
(DOC)

Table S4 Mutations in the global regulators *mucA*, *algU*, *lasR*, and *rpoN* in CFA and CFD isolates.
(DOC)

Table S5 Strains, plasmids, and primers used in this study.
(DOC)

Table S6 Numbers of differentially mutated genes in the CFA and CFD genomes used in Minimum Spanning Trees (MSTs).
(DOC)

Table S7 Average area beneath the respiration curve of reduced tetrazolium vs. time across all the PM1 and PM3B substrates for each CFA and CFD isolate.
(XLS)

Text S1 Analysis of *mutS* and *mutL* gene mutations in the CFA and CFD lineages.
(DOC)
