# Supplementary figures and images for: Coexistence and Within-Host Evolution of Diversified Lineages of Hypermutable Pseudomonas aeruginosa in Long-term Cystic Fibrosis Infections
Source: PLoS Genet. 2014 Oct 16;10(10):e1004651. doi: 10.1371/journal.pgen.1004651 (PMC4199492; doi:10.1371/journal.pgen.1004651)

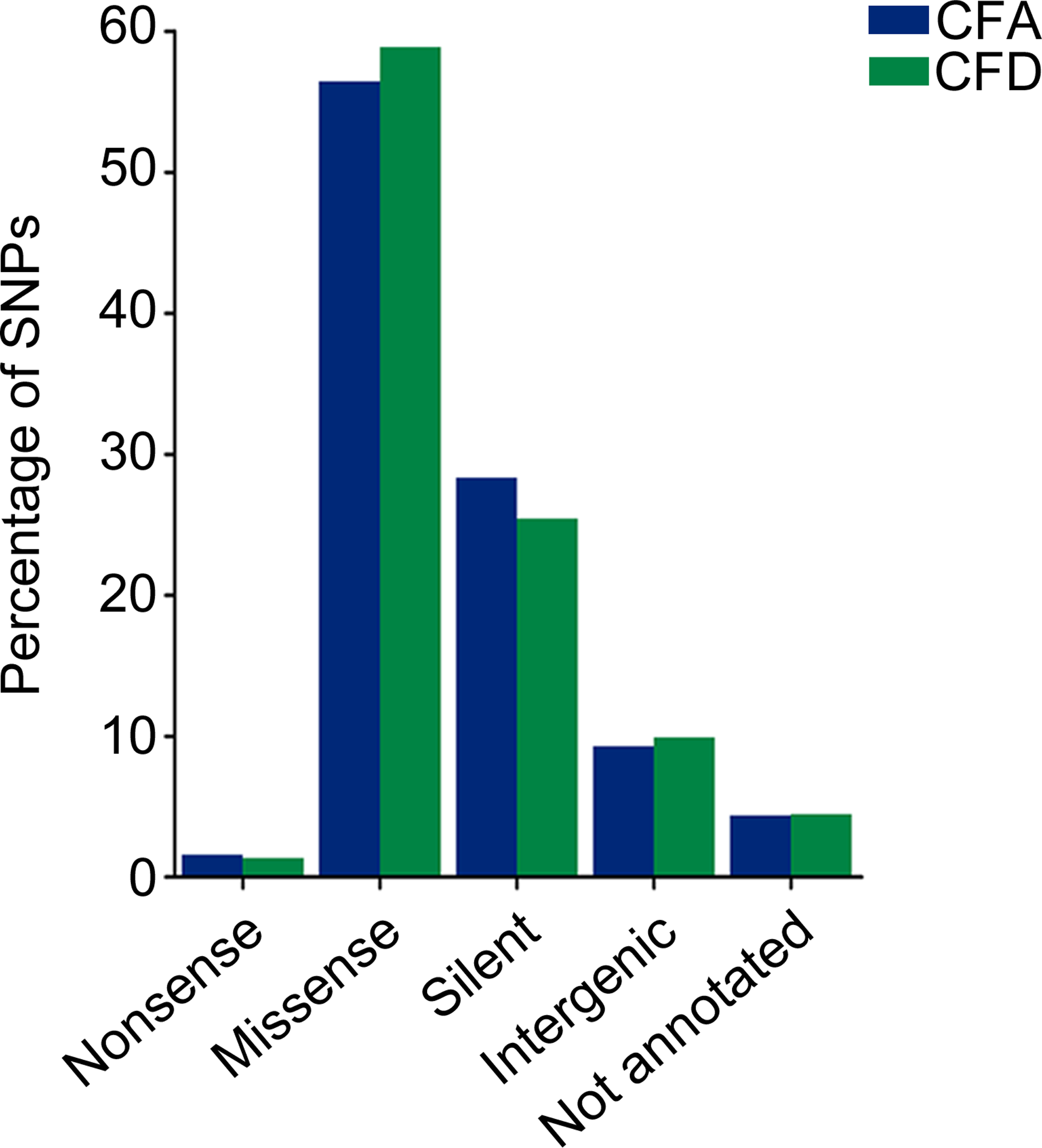

Supplement: Figure S1 — SNP mutations in CFA and CFD lineages. SNPs were analyzed in terms of percentages of nonsense, missense, silent, intergenic, and non-annotated SNPs. (TIF) [file pgen.1004651.s001.tif]

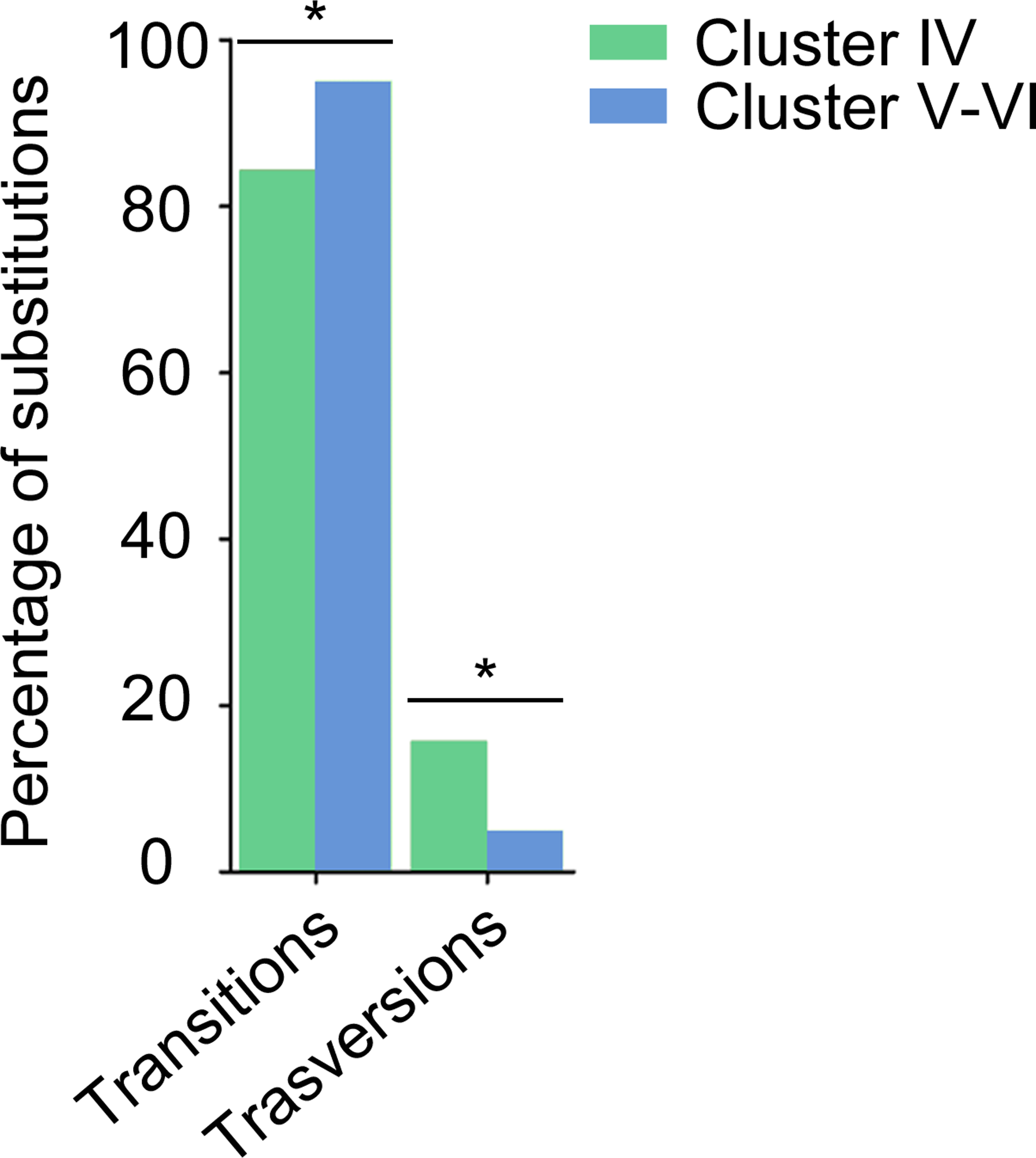

Supplement: Figure S2 — Mutational spectra of CFD normo-mutable and mutator genomes. SNPs were analyzed in terms of the percentages of transitions and transversions in Clusters IV (normo-mutators) and V–VI (mutators). The total number of SNPs from CFD maximum-parsimony phylogenetic tree branches J-I-L-P-O-N-K-M and S-U-T-W-V-X-Q-R-G were considered for Clusters IV and V–VI, respectively (see Figure 2). *: significant difference (P<0.05). (TIF) [file pgen.1004651.s002.tif]
