# Supplementary material for: Coexistence and Within-Host Evolution of Diversified Lineages of Hypermutable Pseudomonas aeruginosa in Long-term Cystic Fibrosis Infections
Source: PLoS Genet. 2014 Oct 16;10(10):e1004651. doi: 10.1371/journal.pgen.1004651 (PMC4199492; doi:10.1371/journal.pgen.1004651)
Supplement: Table S1 — De novo assemblies of P. aeruginosa CFA_2004/01 and CFD_1991/01 genomes. (DOC) [file pgen.1004651.s003.doc]

**Table S1.** *De novo* assemblies of *P. aeruginosa* CFA_2004/01 and CFD_1991/01 genomes.

| **Isolate** | **Reads generated** | **Reads assembled** | **Genome size** | **Coverage depths^a^** | **N° of contigs** | **GC content (%)** | **Reads mapped against strain PAO1 (%)** |
| --- | --- | --- | --- | --- | --- | --- | --- |
| CFA_2004/01 | 12936678 | 12344979 | 6294248 | 172.3 | 844 | 66.3 | 93.9 |
| CFD_1991/01 | 14633218 | 14540799 | 6313855 | 207.2 | 601 | 66.6 | 94.3 |

^a^Coverage depths were calculated for covered positions only.
