# Supplementary material for: Coexistence and Within-Host Evolution of Diversified Lineages of Hypermutable Pseudomonas aeruginosa in Long-term Cystic Fibrosis Infections
Source: PLoS Genet. 2014 Oct 16;10(10):e1004651. doi: 10.1371/journal.pgen.1004651 (PMC4199492; doi:10.1371/journal.pgen.1004651)
Supplement: Table S2 — Illumina sequencing and mapping statistics. (DOC) [file pgen.1004651.s004.doc]

**Table S2.** Illumina sequencing and mapping statistics.

| **Isolate** | | **Read sequences** | **Aligned reads^a^** | **Coverage depths^b^** |
| --- | --- | --- | --- | --- |
| CFA | 2007/01 | 11127570 | 10841829 | 155.611 |
|  | 2010/40 | 8212026 | 7539846 | 106.917 |
|  | 2010/31 | 7877306 | 7523401 | 107.417 |
|  | 2010/01 | 8135826 | 7210409 | 101.4 |
|  | 2010/78 | 7151856 | 6735908 | 95.795 |
|  | 2010/82 | 8049138 | 7612194 | 108.081 |
|  | 2010/43 | 6957194 | 5952189 | 82.8091 |
|  | 2010/72 | 8040018 | 7436158 | 105.449 |
|  | 2010/87 | 8139510 | 7635212 | 108.746 |
|  | 2010/32 | 8199772 | 7967148 | 113.96 |
|  | 2010/26 | 8428428 | 8175018 | 116.864 |
|  | 2010/11 | 7808846 | 7466590 | 106.691 |
| CFD | 2011/33 | 7390194 | 7113516 | 101.786 |
|  | 2002/01 | 8314242 | 8117964 | 117.075 |
|  | 1995/01 | 8390310 | 7692706 | 108.662 |
|  | 2011/95 | 10324514 | 9851342 | 139.745 |
|  | 2011/04 | 7813744 | 7658975 | 109.752 |
|  | 2011/45 | 6234390 | 6001127 | 85.3484 |
|  | 2011/11 | 8934472 | 8647121 | 123.507 |
|  | 2011/57 | 9097206 | 8503838 | 120.671 |
|  | 2011/83 | 8473348 | 8093306 | 101.756 |
|  | 2011/27 | 3805430 | 3616897 | 51.6413 |
|  | 2011/34 | 6928696 | 6547821 | 93.3256 |
|  | 2011/28 | 8639182 | 8271438 | 118.062 |
|  | 2011/94 | 4500106 | 4327669 | 32.6189 |

^a^Reads were mapped against reference genomes of either CFA_2004/01 or CFD_1991/01.

^b^Coverage depths were calculated for covered positions only.

^c^CFA_2004/01 and CFD_1991/01 reference genomes comprise 6,294,248 and 6,313,855 bp respectively.
