# Supplementary material for: Coexistence and Within-Host Evolution of Diversified Lineages of Hypermutable Pseudomonas aeruginosa in Long-term Cystic Fibrosis Infections
Source: PLoS Genet. 2014 Oct 16;10(10):e1004651. doi: 10.1371/journal.pgen.1004651 (PMC4199492; doi:10.1371/journal.pgen.1004651)
Supplement: Table S3 — Numbers of total SNPs and indels in the sequenced genomes. (DOC) [file pgen.1004651.s005.doc]

**Table S3.** Numbers of total SNPs and indels in the sequenced genomes.

| **Genome** | | **Total SNPs^a^** | **Total indels^b^** | **Genome** | | **Total SNPs^c^** | **Total indels^d^** |
| --- | --- | --- | --- | --- | --- | --- | --- |
| CFA | 2007/01 | 294 | 72 | CFD | 2011/33 | 1103 | 202 |
|  | 2010/40 | 1080 | 209 |  | 2002/01 | 889 | 163 |
|  | 2010/31 | 678 | 161 |  | 1995/01 | 629 | 128 |
|  | 2010/01 | 680 | 160 |  | 2011/95 | 1885 | 309 |
|  | 2010/78 | 660 | 160 |  | 2011/04 | 1883 | 294 |
|  | 2010/82 | 517 | 106 |  | 2011/45 | 1886 | 304 |
|  | 2010/43 | 520 | 108 |  | 2011/11 | 1882 | 294 |
|  | 2010/72 | 515 | 104 |  | 2011/57 | 1885 | 307 |
|  | 2010/87 | 390 | 101 |  | 2011/83 | 1433 | 263 |
|  | 2010/32 | 452 | 120 |  | 2011/27 | 1537 | 229 |
|  | 2010/26 | 461 | 131 |  | 2011/34 | 1505 | 241 |
|  | 2010/11 | 459 | 125 |  | 2011/28 | 1718 | 273 |
|  |  |  |  |  | 2011/94 | 1592 | 222 |

^a,b,c,d^SNPs and microindels (1-10 bp) were analyzed as the accumulation of mutations against CFA_2004/01 and CFD_1991/01 reference genomes, as appropriate. Total unique SNPs accumulated: 2578 (CFA) and 5710 (CFD). Total unique microindels accumulated: 544 (CFA) and 1078 (CFD).
