# Supplementary material for: Coexistence and Within-Host Evolution of Diversified Lineages of Hypermutable Pseudomonas aeruginosa in Long-term Cystic Fibrosis Infections
Source: PLoS Genet. 2014 Oct 16;10(10):e1004651. doi: 10.1371/journal.pgen.1004651 (PMC4199492; doi:10.1371/journal.pgen.1004651)
Supplement: Table S4 — Mutations in the global regulators mucA, algU, lasR, and rpoN in CFA and CFD isolates. (DOC) [file pgen.1004651.s006.doc]

Table S4. Mutations in the global regulators *mucA*, *algU*, *lasR*, and *rpoN* in CFA and CFD isolates.

| **Isolate** | | **Mutations^a^** | | | |
| --- | --- | --- | --- | --- | --- |
|  |  | ***mucA*** | ***algU*** | ***lasR*** | ***rpoN*** |
| CFA | 2004/01 | -G a 426 | NF | NF | NF |
|  | 2007/01 | -G a 426 | NF | NF | NF |
|  | 2010/40 | -G a 426 | NF | A343G (T115A) | NF |
|  | 2010/31 | -G a 426 | NF | NF | NF |
|  | 2010/01 | -G a 426 | NF | NF | NF |
|  | 2010/78 | -G a 426 | NF | NF | NF |
|  | 2010/82 | -G a 426 | A170G (K57R) | C224T (T75M) | NF |
|  | 2010/43 | -G a 426 | A170G (K57R) | C224T (T75M) | NF |
|  | 2010/72 | -G a 426 | A170G (K57R) | C224T (T75M) | NF |
|  | 2010/87 | -G a 426 | A176G (Y59C) | NF | NF |
|  | 2010/32 | -G a 426 | A134G (Q45R) | A14G (D5G); C284T (T95M) | NF |
|  | 2010/26 | -G a 426 | A134G (Q45R) | A14G (D5G); C284T (T95M) | NF |
|  | 2010/11 | -G a 426 | A134G (Q45R) | A14G (D5G); C284T (T95M) | NF |
| CFD | 1991/01 | NF | NF | G314A (A105T) | A884G (D295G) |
|  | 2011/33 | NF | NF | G314A (A105T) | A884G (D295G) |
|  | 2002/01 | NF | NF | G314A (A105T) | A884G (D295G) |
|  | 1995/01 | NF | NF | G314A (A105T) | A884G (D295G) |
|  | 2011/95 | A358G (T120A) | NF | G314A (A105T) | A884G (D295G) |
|  | 2011/04 | A358G (T120A) | NF | G314A (A105T) | A884G (D295G) |
|  | 2011/45 | A358G (T120A) | NF | G314A (A105T) | A884G (D295G) |
|  | 2011/11 | A358G (T120A) | NF | G314A (A105T) | A884G (D295G) |
|  | 2011/57 | A358G (T120A) | NF | G314A (A105T) | A884G (D295G) |
|  | 2011/83 | NF | NF | G314A (A105T) | A884G (D295G) |
|  | 2011/27 | NF | NF | G314A (A105T) | A884G (D295G) |
|  | 2011/34 | NF | NF | G314A (A105T) | A884G (D295G) |
|  | 2011/28 | NF | NF | G314A (A105T) | A884G (D295G) |
|  | 2011/94 | NF | NF | G314A (A105T) | A884G (D295G) |

^a^SNPs and indels (1-10 bp) were considered. Amino acid changes are shown in parentheses. NF: no mutations found. -: deletion.
