# Supplementary material for: Coexistence and Within-Host Evolution of Diversified Lineages of Hypermutable Pseudomonas aeruginosa in Long-term Cystic Fibrosis Infections
Source: PLoS Genet. 2014 Oct 16;10(10):e1004651. doi: 10.1371/journal.pgen.1004651 (PMC4199492; doi:10.1371/journal.pgen.1004651)
Supplement: Table S5 — Strains, plasmids, and primers used in this study. (DOC) [file pgen.1004651.s007.doc]

**Table S5.** Strains, plasmids, and primers used in this study.

|  | **Genotype, relevant characteristics, or sequence (5’- 3’)** | **Source or reference** |
| --- | --- | --- |
| **Strains** |  |  |
| *P. aeruginosa* |  |  |
| PAO1 | wild-type; phototropic | [1] |
| MPAO1MS | *mutS::ISlacZA/hah* (MPA32417); Tc^r^ | [2] |
| MPAO1ML | *mutL::ISlacZA/hah* (MPA46306); Tc^r^ | [2] |
| *E. coli* |  |  |
| *E. coli* Dh5α | Host for DNA manipulation | Invitrogen |
| **Plasmids** |  |  |
| pMC5-MutS | pBBR1MCS-5 carrying *P.aeruginosa mutS*; Gm^r^ | [3] |
| pMC5-MutL | pBBR1MCS-5 carrying *P.aeruginosa mutL*; Gm^r^ | [4] |
| pMC5-MutS-CG_1551_ | pBBR1MCS-5 carrying CFD_2011/27 *mutS*; Gm^r^ | This study |
| pMC5-MutS+CC_334_-CG_1551_ | pBBR1MCS-5 carrying CFD_2011/11 *mutS*; Gm^r^ | This study |
| **Primers** |  |  |
| 272 | AGCGGGCCAA | [5] |
| *mutS*-for1 | GCCCGTATGACCGACCTCT | [6] |
| *mutS*-rev1 | CCGAGTCGCGATCGAAGT | [6] |
| *mutS*-for2 | CCGCGCGCCATGGGACTTCGAT | [6] |
| *mutS*-rev2 | TTCGGCGAGTTCGGGATA | [6] |
| *mutS*-for3 | CACCACCATCGGCACCTAT | [6] |
| *mutS*-rev3 | GTTGGCCACGAACGGTGT | [6] |
| *mutS*-for4 | TGGTCGAGCAGGTGCTGG | [6] |
| *mutS*-rev4 | ATTCTAGCAGCTTGTGCGG | [6] |
| *mutL*-for1 | ACAGCCTGTCCAGCGACAAC | [6] |
| *mutL*-rev1 | CTCGTCTCGCGCCTCGTGCA | [6] |
| *mutL*-for2 | TGCACGAGGCGCGAGACGAG | [6] |
| *mutL*-rev2 | TAACGGCGCGAAGTAGGCCTT | [6] |
| *mutL*-for3 | AAGGCCTACTTCGCGCCGTTA | [6] |
| *mutL*-rev3 | AGGCAGGGAAGACATCGGAAC | [6] |

Tc^r^: tetracycline resistance. Gm^r^: gentamicin resistance.

**References**

1. Holloway BW (1955) Genetic recombination in *Pseudomonas aeruginosa*. J Gen Microbiol 13: 572-581.

2. Jacobs MA, Alwood A, Thaipisuttikul I, Spencer D, Haugen E, et al. (2003) Comprehensive transposon mutant library of *Pseudomonas aeruginosa*. Proc Natl Acad Sci U S A 100: 14339-14344.

3. Pezza RJ, Smania AM, Barra JL, Argaraña CE (2002) Nucleotides and heteroduplex DNA preserve the active conformation of *Pseudomonas aeruginosa* MutS by preventing protein oligomerization. Biochem J 361: 87-95.

4. Jacquelín DK, Filiberti A, Argaraña CE, Barra JL (2005) *Pseudomonas aeruginosa* MutL protein functions in *Escherichia coli*. Biochem J 388: 879-887.

5. Mahenthiralingam E, Campbell ME, Foster J, Lam JS, Speert DP (1996) Random amplified polymorphic DNA typing of *Pseudomonas aeruginosa* isolates recovered from patients with cystic fibrosis. J Clin Microbiol 34: 1129-1135.

6. Feliziani S, Luján AM, Moyano AJ, Sola C, Bocco JL, et al. (2010) Mucoidy, quorum sensing, mismatch repair and antibiotic resistance in *Pseudomonas aeruginosa* from cystic fibrosis chronic airways infections. PLoS One 5.
