# Supplementary material for: Coexistence and Within-Host Evolution of Diversified Lineages of Hypermutable Pseudomonas aeruginosa in Long-term Cystic Fibrosis Infections
Source: PLoS Genet. 2014 Oct 16;10(10):e1004651. doi: 10.1371/journal.pgen.1004651 (PMC4199492; doi:10.1371/journal.pgen.1004651)
Supplement: Table S6 — Numbers of differentially mutated genes in the CFA and CFD genomes used in Minimum Spanning Trees (MSTs). (DOC) [file pgen.1004651.s008.doc]

**Table S6.** Numbers of differentially mutated genes in the CFA and CFD genomes used in Minimum Spanning Trees (MSTs).

|  | | **CFA** | | | | | | | | | | | | | |
| --- | --- | --- | --- | --- | --- | --- | --- | --- | --- | --- | --- | --- | --- | --- | --- |
|  |  | 2004/01 | 2007/01 | 2010/01 | 2010/11 | 2010/26 | 2010/31 | 2010/32 | 2010/40 | 2010/43 | 2010/72 | 2010/78 | 2010/82 | 2010/87 |  |
| **CFA** | 2004/01 | 0 |  |  |  |  |  |  |  |  |  |  |  |  |  |
|  | 2007/01 | 198 | 0 |  |  |  |  |  |  |  |  |  |  |  |  |
|  | 2010/01 | 450 | 472 | 0 |  |  |  |  |  |  |  |  |  |  |  |
|  | 2010/11 | 318 | 344 | 558 | 0 |  |  |  |  |  |  |  |  |  |  |
|  | 2010/26 | 321 | 343 | 555 | 15 | 0 |  |  |  |  |  |  |  |  |  |
|  | 2010/31 | 456 | 480 | 78 | 568 | 567 | 0 |  |  |  |  |  |  |  |  |
|  | 2010/32 | 317 | 337 | 553 | 11 | 10 | 563 | 0 |  |  |  |  |  |  |  |
|  | 2010/40 | 673 | 683 | 769 | 757 | 754 | 779 | 750 | 0 |  |  |  |  |  |  |
|  | 2010/43 | 350 | 374 | 588 | 306 | 305 | 596 | 303 | 763 | 0 |  |  |  |  |  |
|  | 2010/72 | 355 | 377 | 591 | 311 | 308 | 599 | 306 | 766 | 7 | 0 |  |  |  |  |
|  | 2010/78 | 445 | 469 | 31 | 551 | 548 | 73 | 546 | 764 | 581 | 584 | 0 |  |  |  |
|  | 2010/82 | 350 | 374 | 588 | 306 | 305 | 596 | 303 | 763 | 8 | 11 | 581 | 0 |  |  |
|  | 2010/87 | 269 | 297 | 505 | 167 | 170 | 513 | 164 | 728 | 267 | 270 | 496 | 265 | 0 |  |
|  | | **CFD** | | | | | | | | | | | | | |
|  |  | 1991/01 | 1995/01 | 2002/01 | 2011/04 | 2011/11 | 2011/27 | 2011/28 | 2011/33 | 2011/34 | 2011/45 | 2011/57 | 2011/83 | 2011/94 | 2011/95 |
| **CFD** | 1991/01 | 0 |  |  |  |  |  |  |  |  |  |  |  |  |  |
|  | 1995/01 | 427 | 0 |  |  |  |  |  |  |  |  |  |  |  |  |
|  | 2002/01 | 580 | 733 | 0 |  |  |  |  |  |  |  |  |  |  |  |
|  | 2011/04 | 1108 | 899 | 1260 | 0 |  |  |  |  |  |  |  |  |  |  |
|  | 2011/11 | 1112 | 899 | 1262 | 40 | 0 |  |  |  |  |  |  |  |  |  |
|  | 2011/27 | 927 | 732 | 1093 | 625 | 629 | 0 |  |  |  |  |  |  |  |  |
|  | 2011/28 | 1025 | 836 | 1159 | 699 | 703 | 546 | 0 |  |  |  |  |  |  |  |
|  | 2011/33 | 724 | 909 | 1000 | 1364 | 1364 | 1263 | 1285 | 0 |  |  |  |  |  |  |
|  | 2011/34 | 912 | 721 | 1074 | 614 | 614 | 183 | 533 | 1234 | 0 |  |  |  |  |  |
|  | 2011/45 | 1121 | 908 | 1261 | 43 | 43 | 632 | 712 | 1375 | 613 | 0 |  |  |  |  |
|  | 2011/57 | 1117 | 900 | 1261 | 37 | 35 | 626 | 706 | 1365 | 609 | 36 | 0 |  |  |  |
|  | 2011/83 | 892 | 693 | 1032 | 618 | 622 | 351 | 503 | 1226 | 320 | 625 | 613 | 0 |  |  |
|  | 2011/94 | 974 | 795 | 1122 | 684 | 688 | 529 | 65 | 1264 | 508 | 701 | 695 | 480 | 0 |  |
|  | 2011/95 | 1115 | 904 | 1261 | 49 | 51 | 632 | 704 | 1369 | 617 | 52 | 44 | 625 | 693 | 0 |
