# Supplementary material for: Coexistence and Within-Host Evolution of Diversified Lineages of Hypermutable Pseudomonas aeruginosa in Long-term Cystic Fibrosis Infections
Source: PLoS Genet. 2014 Oct 16;10(10):e1004651. doi: 10.1371/journal.pgen.1004651 (PMC4199492; doi:10.1371/journal.pgen.1004651)
Supplement: Text S1 — Analysis of mutS and mutL gene mutations in the CFA and CFD lineages. (DOC) [file pgen.1004651.s010.doc]

**Text S1**

**Analysis of *mutS* and *mutL* gene mutations in the CFA and CFD lineages**

We evaluated the genetic bases for the mutator phenotypes of the 27 *P. aeruginosa* CFA and CFD isolates by gene complementation assays and by sequence analysis of *mutS* and *mutL* genes. CFA mutator isolates harbored different missense mutations in both MRS genes (Table 1). The mutations observed in the *mutS* and *mutL* coding sequences were alternatively present in the CFA isolates, generating three different *mutS/L* allelic combinations (SL1-3). One mutation in *mutS* (A739C) and one in *mutL* (A1406G) were shared by all CFA clones. Genetic complementation showed that the A1406G mutation in *mutL* but not the A739C mutation in *mutS* produced the loss of function of the gene product, suggesting that this *mutL* allele caused the mutator phenotype. The A1406G mutation causes a change in amino acid 469 from histidine to arginine (H469R). H469, together with D467 and E473, is part of a highly conserved motif (DMHAAHERITYE) that is important for MutL endonuclease activity. This enzyme activity is essential for *in vivo* MutL and mismatch repair functions not only in *P. aeruginosa* [1] but also in yeast and humans [2,3,4].

A739C is located in the connector domain of the MutS protein. A1406G-*mutL*/A739C-*mutS* mutations generate the SL1 allelic combination, which is prevalent in the CFA population (Table 1). From the A1406G-*mutL*/A739C-*mutS* background, the SL2 and SL3 allelic combinations are composed by *mutS* mutations T478A and T2381C, respectively (Table 1). Both of these mutations are located in the connector and the helix-turn-helix domains of MutS. None of these *mutS* mutations accounted for the MRS inactivation, because DNA repair functions in CFA isolates that harbored SL2 and SL3 were complemented with the sole plasmid-borne expression of the *mutL* wild-type gene.

All isolates obtained from patient CFD harbored a -CG deletion at 1551 bp from the ATG of *mutS* (Table 1), which produces a stop codon at 1854 bp. This premature stop in translation results in the loss of 237 amino acids of the C-terminal region of MutS; this region has been linked to protein oligomerization and MutS-MutL interaction [5]. MutS tetramerization in *P. aeruginosa* is important for ATP hydrolysis and DNA binding, and both processes are essential for MutS function *in vivo* [6]. Normal MRS activity was restored in CFD isolates by complementation with a wild-type copy of the *mutS* gene, confirming the loss-of-function effect of the -CG1551 mutation. In addition to the -CG1551 deletion, the subpopulation of CFD isolates that displayed a nonmutator phenotype (Cluster IV) carried an insertion of two C at 334 bp (+CC334) downstream of the ATG of *mutS*, thus constituting a new +CC334-CG1551 *mutS* allele (Table 1). The +CC334 mutation produces a premature stop codon at 347 bp, leading to an N-terminal peptide of 116 amino acids, which corresponds precisely to the MutS domain I involved in DNA binding [7]. The +CC334 insertion produces an ATG codon at 1822 bp, leading to restoration of the frameshift encoding a C-terminal peptide of 248 amino acids, which corresponds to oligomerization and MutS-MutL interaction in functionally essential MutS domains [5,6].

**References**

1. Correa EM, Martina MA, De Tullio L, Argaraña CE, Barra JL (2011) Some amino acids of the *Pseudomonas aeruginosa* MutL D(Q/M)HA(X)(2)E(X)(4)E conserved motif are essential for the *in vivo* function of the protein but not for the *in vitro* endonuclease activity. DNA Repair (Amst) 10: 1106-1113.

2. Erdeniz N, Nguyen M, Deschenes SM, Liskay RM (2007) Mutations affecting a putative MutLalpha endonuclease motif impact multiple mismatch repair functions. DNA Repair (Amst) 6: 1463-1470.

3. Fukui K, Nishida M, Nakagawa N, Masui R, Kuramitsu S (2008) Bound nucleotide controls the endonuclease activity of mismatch repair enzyme MutL. J Biol Chem 283: 12136-12145.

4. Kadyrov FA, Holmes SF, Arana ME, Lukianova OA, O'Donnell M, et al. (2007) *Saccharomyces cerevisiae* MutLalpha is a mismatch repair endonuclease. J Biol Chem 282: 37181-37190.

5. Wu TH, Marinus MG (1999) Deletion mutation analysis of the *mutS* gene in *Escherichia coli*. J Biol Chem 274: 5948-5952.

6. Miguel V, Monti MR, Argaraña CE (2008) The role of MutS oligomers on *Pseudomonas aeruginosa* mismatch repair system activity. DNA Repair (Amst) 7: 1799-1808.

7. Obmolova G, Ban C, Hsieh P, Yang W (2000) Crystal structures of mismatch repair protein MutS and its complex with a substrate DNA. Nature 407: 703-710.
